# Supplementary material for: A click chemistry-mediated all-peptide cell printing hydrogel platform for diabetic wound healing
Source: Nat Commun. 2023 Nov 29;14:7856. doi: 10.1038/s41467-023-43364-2 (PMC10687272; doi:10.1038/s41467-023-43364-2)
Supplement: Supplementary file 1 — Supplementary Information [file 41467_2023_43364_MOESM1_ESM.pdf]

# *Supplementary Information*

**A Click Chemistry-mediated All-peptide Cell Printing Hydrogel Platform for Diabetic Wound Healing**

Jinjian Huang, Rong Yang, Jiao Jiao, Ze Li, Penghui Wang, Ye Liu, Sicheng Li, Canwen Chen, Zongan Li, Guiwen Qu, Kang Chen, Xiuwen Wu, Bo Chi, Jianan Ren

**Correspondence to:** Xiuwen Wu, e-mail: wuxiuwen@nju.edu.cn; Bo Chi, e-mail: chibo@njtech.edu.cn; Jianan Ren, e-mail: JiananR@nju.edu.cn.

.

**These authors contributed equally to this work:** Jinjian Huang, Rong Yang, and Jiao Jiao.

**This PDF file includes:**

Supplementary Notes 1 and 2;

Supplementary Figures 1 to 14;

Supplementary Tables 1 and 2;

Supplementary References.

**Supplementary Note 1**

**$^1$H NMR spectrum.** γ-PGA, GMA, Cys, γ-PGA-SH, γ-PGA-GMA, RGDC, and γ-PGA-GMA-RGDC were dissolved in deuterated solvents individually. The modification of γ-PGA and the purity of modified polymers in final products were characterized by $^1$H NMR (Bruker 500, Germany).

**FTIR spectrum.** The powders of tested polymers were ground to a dry KBr disk. 32 scans at a resolution of 4 $cm^{-1}$ were used to record the spectra in the wave number range of 4000–500 $cm^{-1}$ using a Nicolet-6700 spectrometer (Thermo Fisher, USA).

**Swelling ratio.** All prepared hydrogels were weighted ($W_0$) before immersing into 10 mL PBS at 37 °C. In different time intervals, the hydrogels were taken out of PBS and weighed after removing the superficial water on the surface of hydrogels using a filter paper. The experiments were performed in triplicate and the swelling ratio was calculated as the following formula [1]:

$$\text{Swelling ratio} = (W_t - W_0) / W_0 \times 100\%.$$

The weight when reaching absorption equilibrium was $W_t$, and the initial weight was $W_0$.

**Degradation.** Hydrogels with the same volume were prepared in 1 mL molds and accurately weighted ($W_1$). Then hydrogels were incubated in 30 mL simulated body fluid (SBF) at constant temperature (37 °C) [2]. The SBF was refreshed every day. The remaining hydrogel samples were taken out at the predetermined time point and the residual weight ($W_r$) was measured. The degradation of hydrogel was judged by the remained mass percentage of hydrogels calculated based on the following formula [3]:

$$\text{Mass remaining} = W_r / W_1 \times 100\%.$$

**Morphological scanning.** The internal morphology of the hydrogels after freeze-dried was observed using a scanning electron microscope (SEM, LEO 1550, LEO, Germany). Before the examination by SEM, the lyophilized hydrogels were broken off and then the sections were sprayed with a gold layer by vacuum sputter.

**Rheological test.** Rheological analysis of hydrogels was conducted between parallel plates with a 25 mm diameter and a 1 mm gap using the rheometer (DHR-2, TA-Instruments, USA) at 37 °C, in terms of the time sweeps (external blue light with on-and-off cycles; 1.0 Hz; 1% strain), strain sweeps (10 Hz; 1–1000% strain), and oscillatory frequency sweeps (0.1–100 Hz; 10% strain).

**Mechanical test.** The capabilities of the hydrogels to compress were measured with a universal material testing machine (MTS, model: CMT2103, USA). We cast hydrogels in the shape of cylinder

with a diameter of 10 mm and the height of 8 mm for compression tests at 20 mm $min^{-1}$. The fracture energy (U) of the hydrogels was calculated using the following equation based on the integral of the area under the compressive stress ($\tau$) – strain ($\varepsilon$) curve [4]:

$$U = \int \tau d\varepsilon.$$

**Supplementary Note 2**

**Western blot procedures.** Proteins from cytosol or mitochondria of the lysed cells were separated by SDS-PAGE and transferred to PVDF membranes. The membranes were then incubated overnight at 4 °C with antibodies against Bax (1:2000, ab32503, Abcam), Bcl2 (1:2000, ab182858, Abcam), Cox iv (1:2000, ab202554; Abcam), Cyt-c (1:5000, ab133504, Abcam), Cleaved casp-3 (1:500, ab32042, Abcam), Pro-casp-3 (1:1000, ab32150, Abcam), and β-actin (1:1000, ab8226, Abcam). Positive signals were scanned using a Tanon-4600 chemiluminescence imaging system (Tanon, Shanghai, China), and were normalized to the corresponding Cox iv band as a mitochondrial loading control or β-actin band as a cytosol loading control.

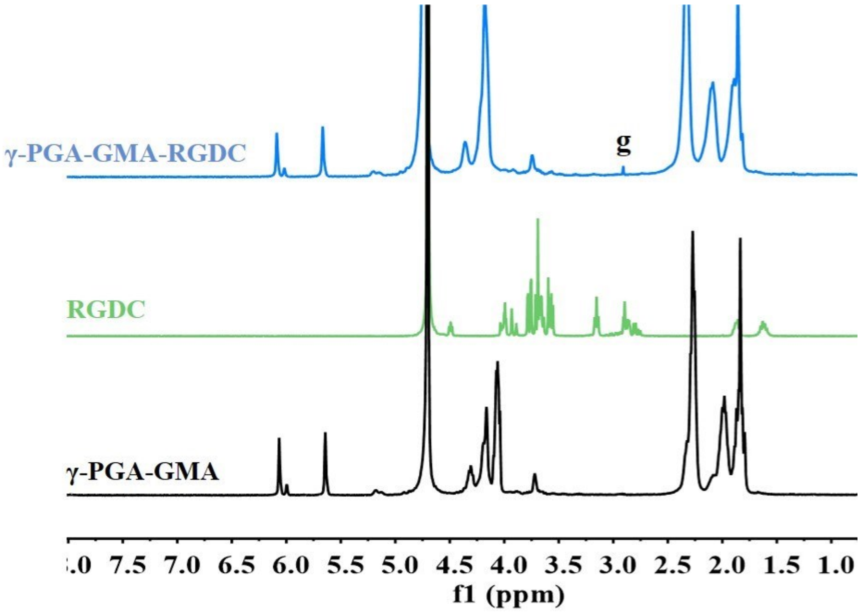


**Supplementary Figure 1.** $^{1}$H NMR spectrum of RGDC-modified γ-PGA-GMA, which confirms the feasibility of RGDC conjugation to the hydrogel via linking with γ-PGA-GMA. g: δ = 2.8–2.9 ppm. Source data are provided as a Source Data file.

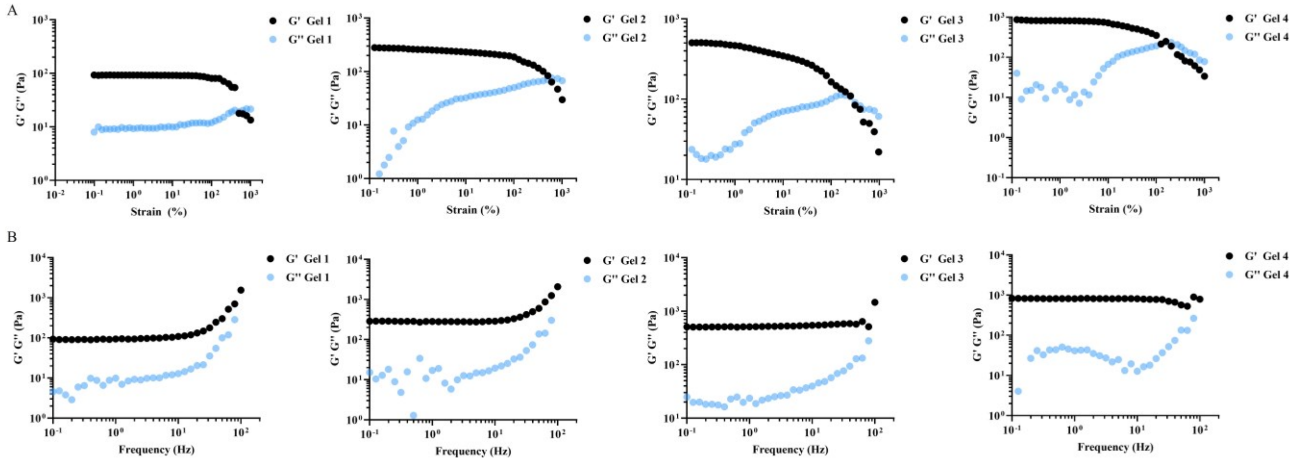


**Supplementary Figure 2.** (A) Strain sweeps (10 Hz; 1–1000% strain) of Gel 1, Gel 2, Gel 3, and Gel 4. (B) Oscillatory frequency sweeps (0.1–100 Hz; 10% strain) of Gel 1, Gel 2, Gel 3, and Gel 4. Source data are provided as a Source Data file.

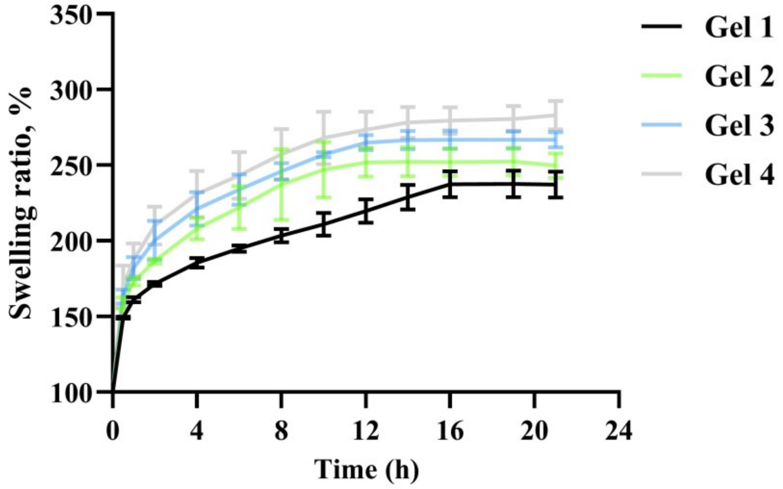


**Supplementary Figure 3.** Swelling ratio of Gel 1, Gel 2, Gel 3, and Gel 4. n = 3 independent experiments. Data are presented as mean ± SD. Source data are provided as a Source Data file.

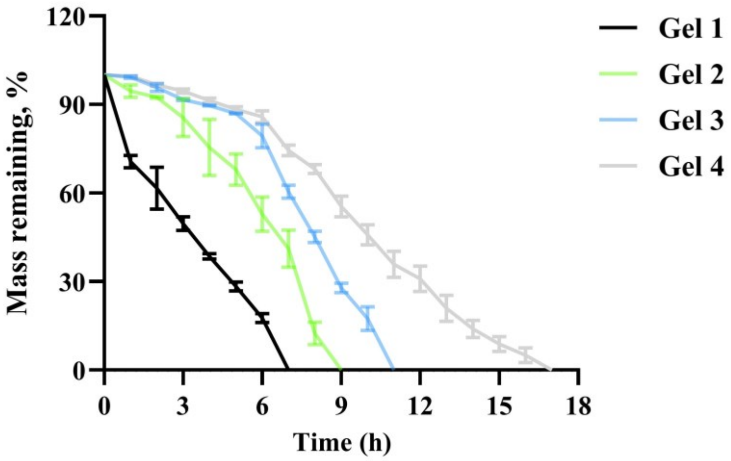


**Supplementary Figure 4.** Degradation of Gel 1, Gel 2, Gel 3, and Gel 4, as judged by the mass remaining percentage in simulated body fluid. n = 3 independent experiments. Data are presented as mean ± SD. Source data are provided as a Source Data file.

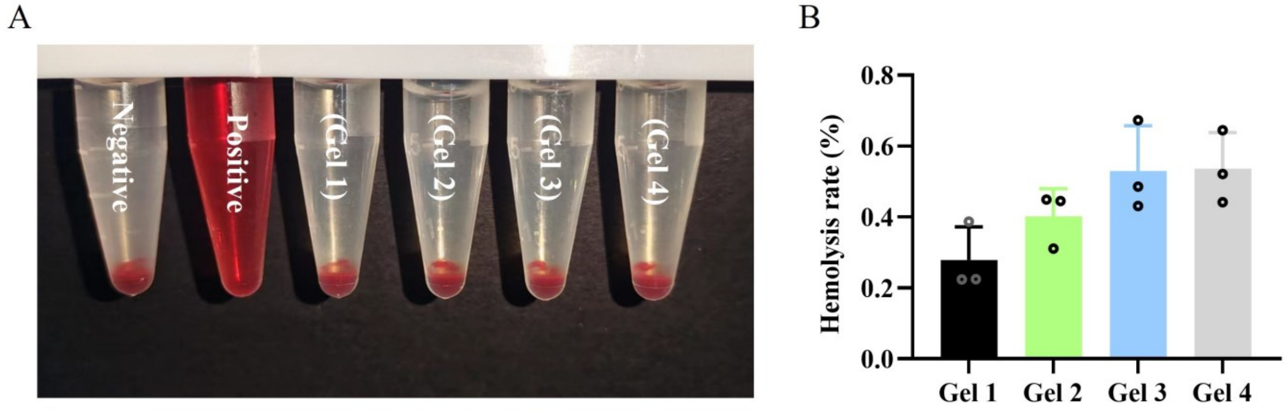


**Supplementary Figure 5.** Cytocompatibility of the all-peptide hydrogels as indicated by (A) the erythrocyte hemolysis, and (B) the hemolysis rate less than 1 %. n = 3 independent experiments. Data are presented as mean ± SD. Source data are provided as a Source Data file.

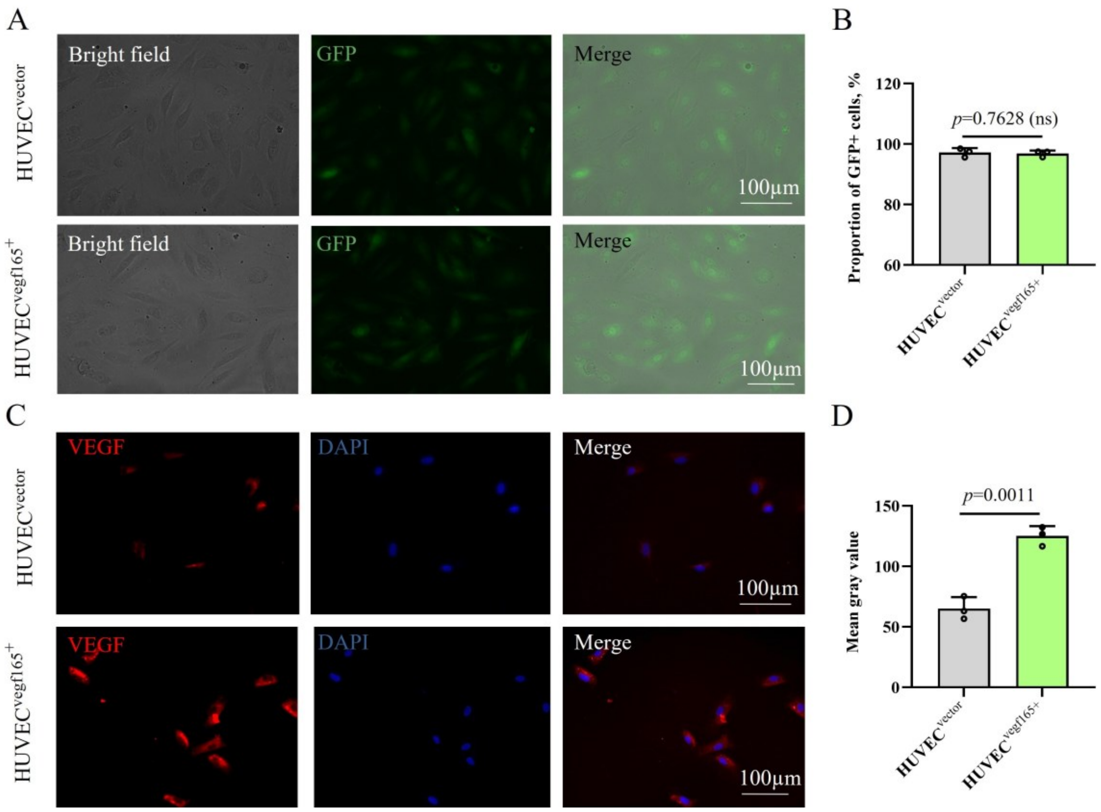


**Supplementary Figure 6.** (A) Images of $GFP^+$ HUVECs transinfected with vector lentivirus or VEGF165-overexpressed lentivirus. (B) Quantitative analysis of $GFP^+$ HUVECs. n = 3 independent experiments. (C) IF staining of VEGF on HUVECs transinfected with vector lentivirus or VEGF165-overexpressed lentivirus. (D) Quantitative analysis of VEGF signals. n = 3 independent experiments. The *p* values in the figures (B and D) are determined by two-sided unpaired *t* test. Data are presented as mean ± SD. ns, not significant. Source data are provided as a Source Data file.

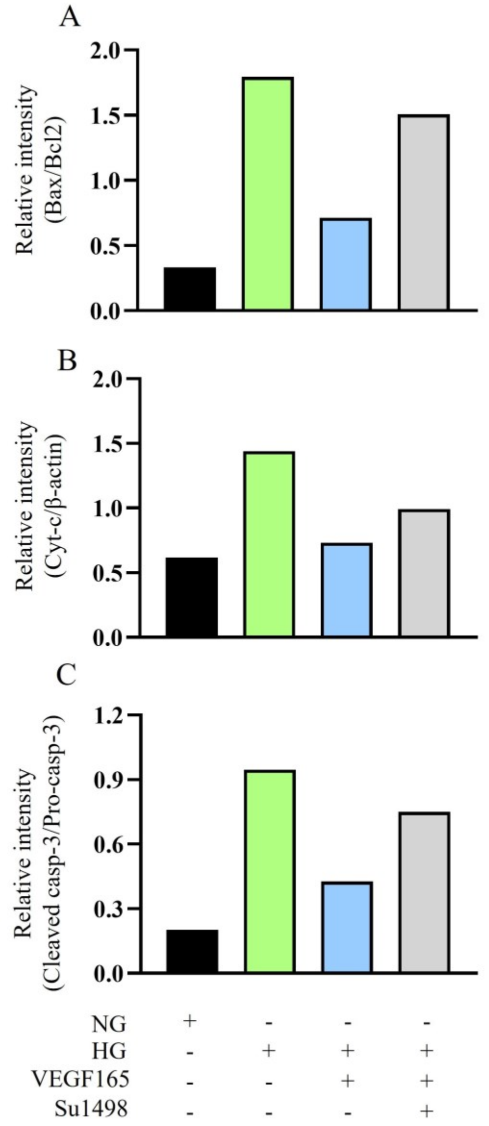


**Supplementary Figure 7.** (A) Ratio of Bax to Bcl2 intensity. (B) Ratio of Cyt-c to β-actin intensity. (C) Ratio of Cleaved casp-3 to pro-casp-3 intensity.

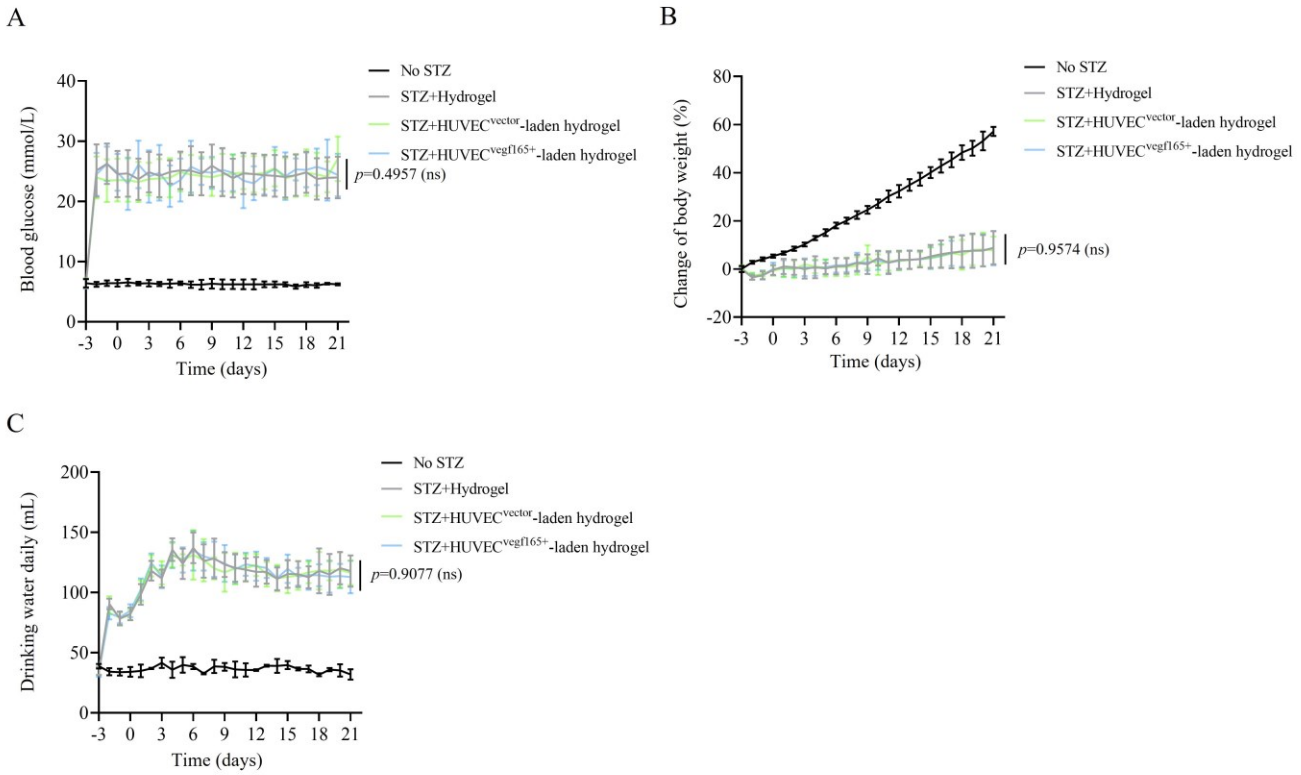


**Supplementary Figure 8.** Successful creation of rat diabetic model dependent on intraperitoneal injection of streptozotocin at a dose of 70 mg/kg, which was confirmed by (A) significant elevation of blood glucose; (B) decrease in body weight; (C) increase in water consumption. The $p$ values in the figures (A-C) that present the treatment effect for the STZ-induced rat diabetic wound model are determined by two-way ANOVA. Data are presented as mean ± SD. ns, not significant. Source data are provided as a Source Data file.

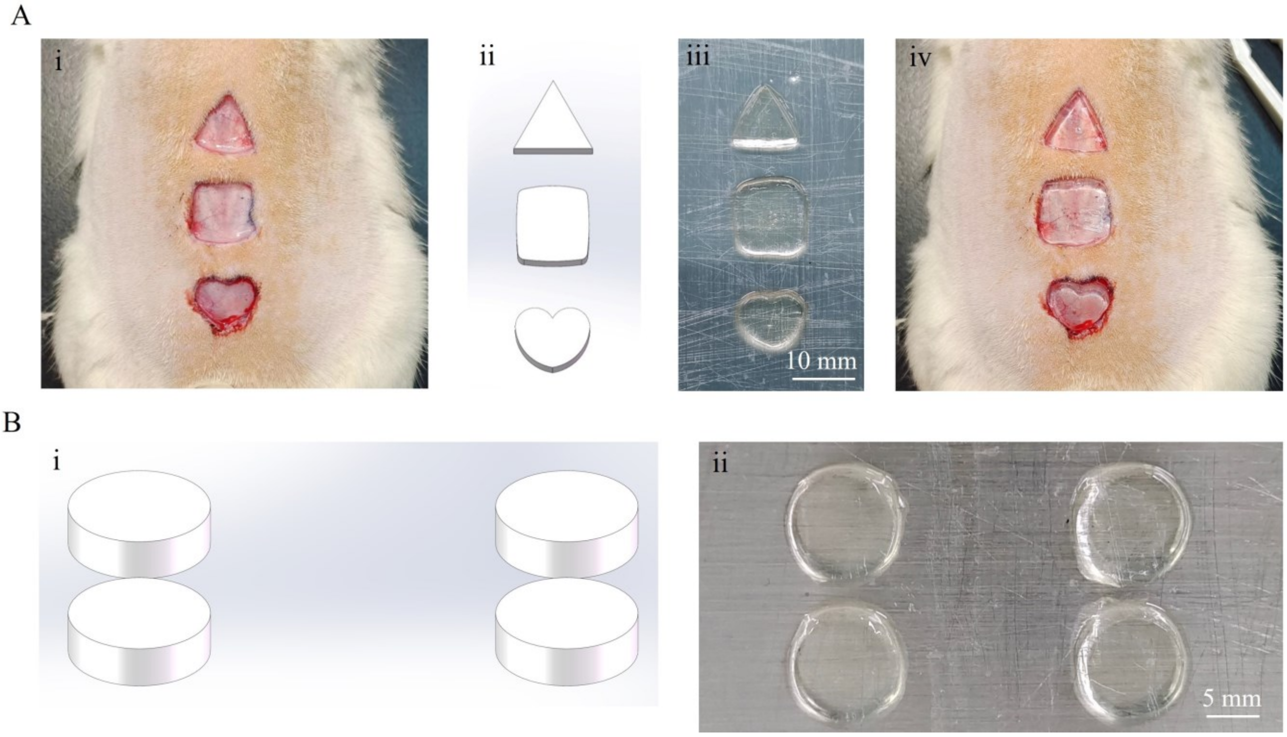


**Supplementary Figure 9.** (A) Shape adaptability for irregular wounds by DLP printing of hydrogel. i: creating wounds in different shapes; ii: designing hydrogel shapes; iii: DLP printing of hydrogels; iv: the shape of printed hydrogels was adaptive to the wounds. (B) High throughput production of DLP printed hydrogels. The example showed that four hydrogels were printed simultaneously. i: designing four hydrogels; ii: DLP printing of the designed hydrogels.

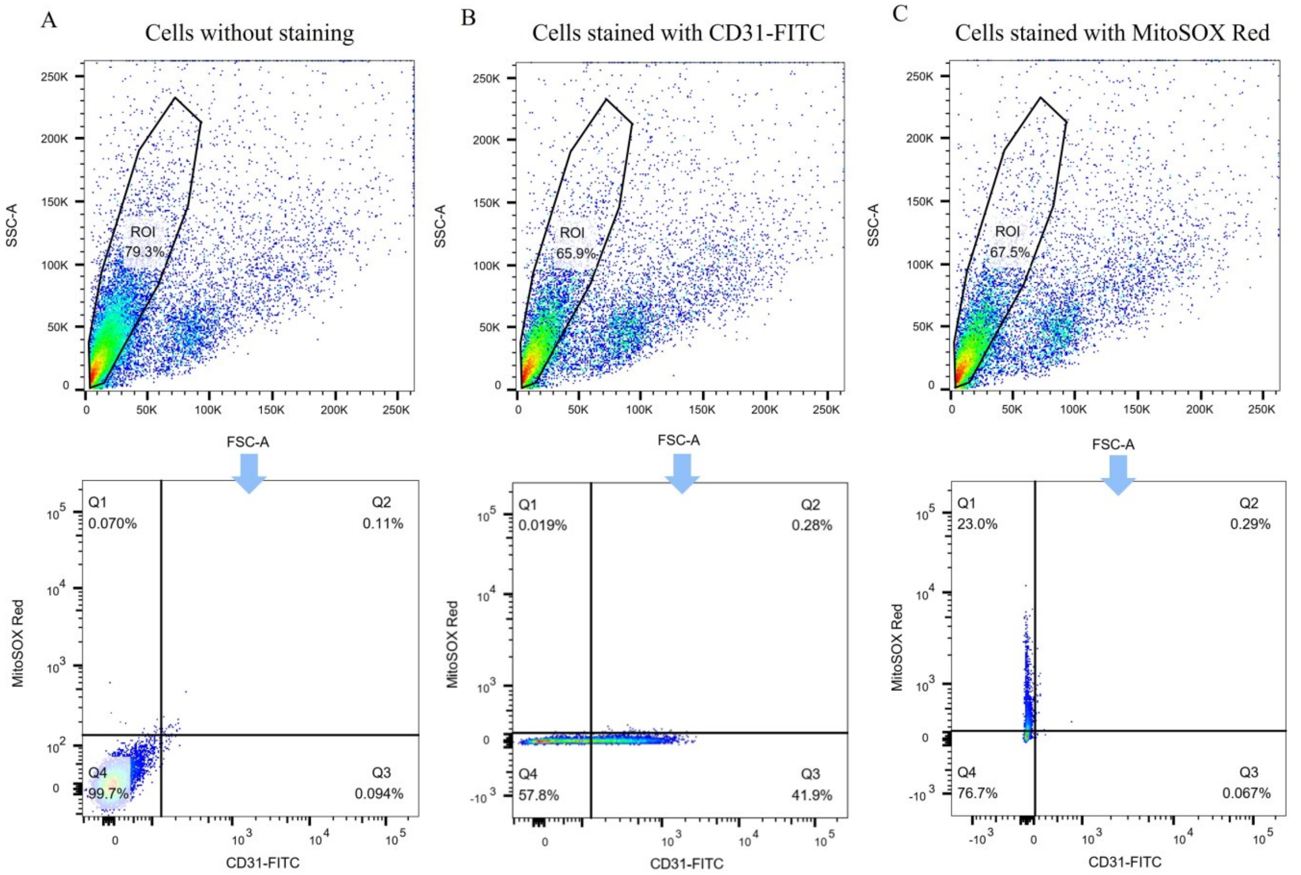


**Supplementary Figure 10.** Verification on the accuracy of flow cytometry gating condition. (A) Under the gating condition, the cells without dye staining were mostly presented in Q4. (B) Under the gating condition, the cells stained merely by CD31-FITC were mostly presented in Q3 and Q4. (C) Under the gating condition, the cells stained merely by MitoSOX Red were mostly presented in Q1 and Q4.

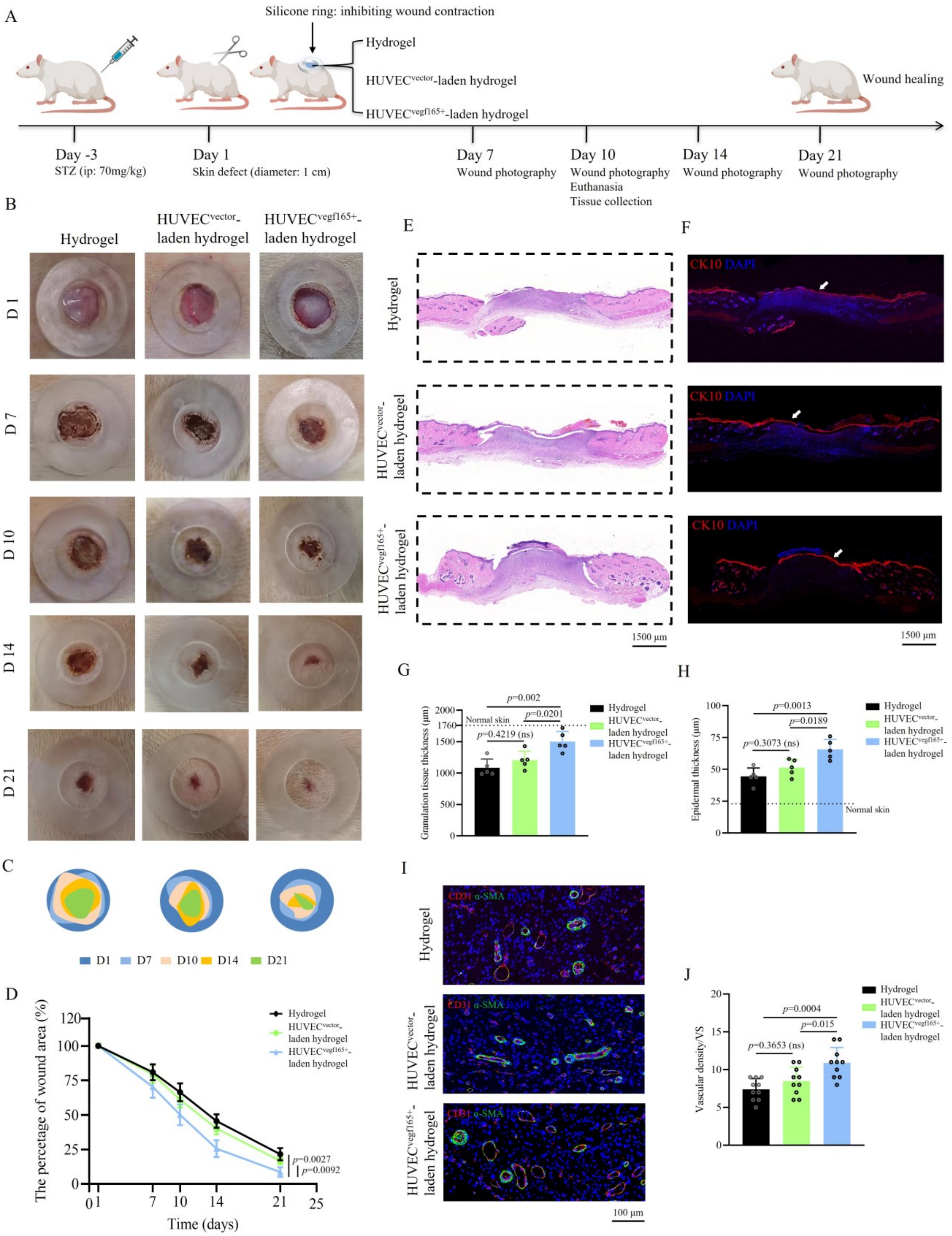


**Supplementary Figure 11.** Evaluation of the effect of HUVEC$^{vegf165+}$-laden hydrogel on rat diabetic wounds whose contraction is inhibited by a silicone ring. (A) Experimental scheme. This figure was created with BioRender.com and has been granted a publication license. ip, intraperitoneal injection. (B) Diabetic wound healing processes were recorded after the different treatments. Inner diameter of

the silicone ring: 1 cm. (C) Re-depiction of wound healing processes. (D) Comparison of wound closure rates following the different treatments. n = 10 rats/group on days 1, 7, and 10; n = 5 rats/group on days 14 and 21. (E) HE analysis revealed varied degrees of granulation tissue formation in the different treatment groups on day 10. (F) CK10 immunofluorescence (IF) staining showed varied degrees of re-epithelization in the different treatment groups on day 10. Arrows: regenerated epithelial layers. (G) Quantitative analysis of granulation tissue thickness in the different treatment groups on day 10. n = 5 rats/group. (H) Quantitative analysis of regenerated epithelial layer thickness in the different treatment groups on day 10. n = 5 rats/group. (I) Representative vascular staining of regenerated granulation tissues in the different treatment groups on day 10. (J) Comparison of vascular density in the different treatment groups on day 10. n = 10 because two random visual fields were selected for each tissue sample harvested from five rats in each group. The *p* values in the figure (D) and figure (G, H, and J) are determined by two-way ANOVA and one-way ANOVA, respectively, followed by Tukey's multiple comparisons test. Data are presented as mean ± SD. ns, not significant. Source data are provided as a Source Data file.

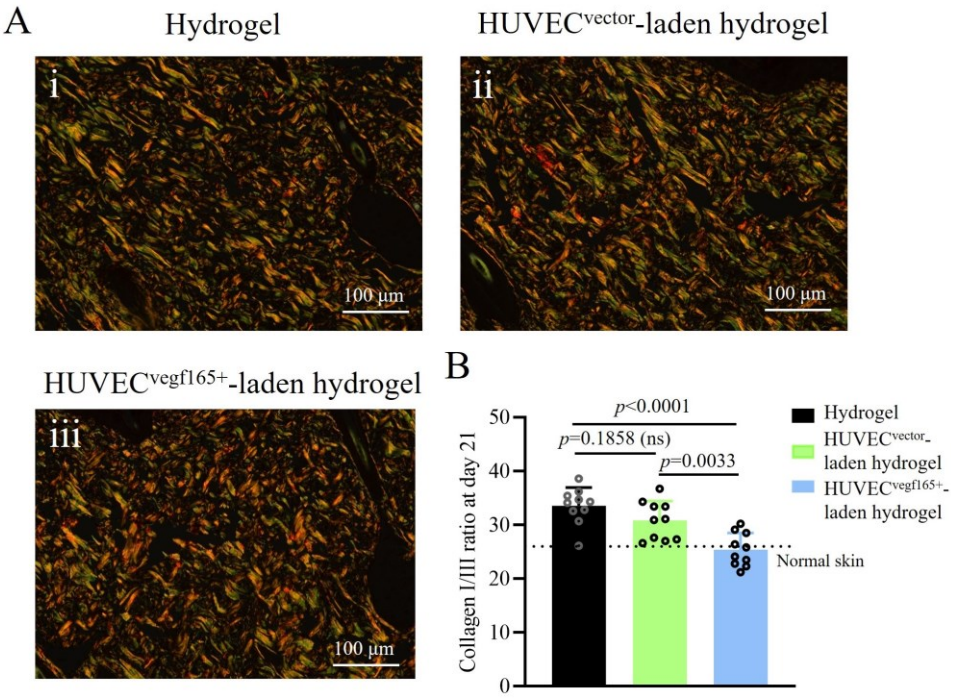


**Supplementary Figure 12.** (A) Picrosirius red staining of collagen in the wounds on day 21. i: treated with hydrogel; ii: treated with HUVEC$^{vector}$-laden hydrogel; iii: treated with HUVEC$^{vegf165+}$-laden hydrogel. (B) Quantitative analysis of collagen I/III ratio on day 21. n = 10 because two random visual fields were selected for each tissue sample harvested from five rats in each group. The *p* values in the figure (B) are determined by one-way ANOVA followed by Tukey's multiple comparisons test. Data are presented as mean ± SD. ns, not significant. Source data are provided as a Source Data file.

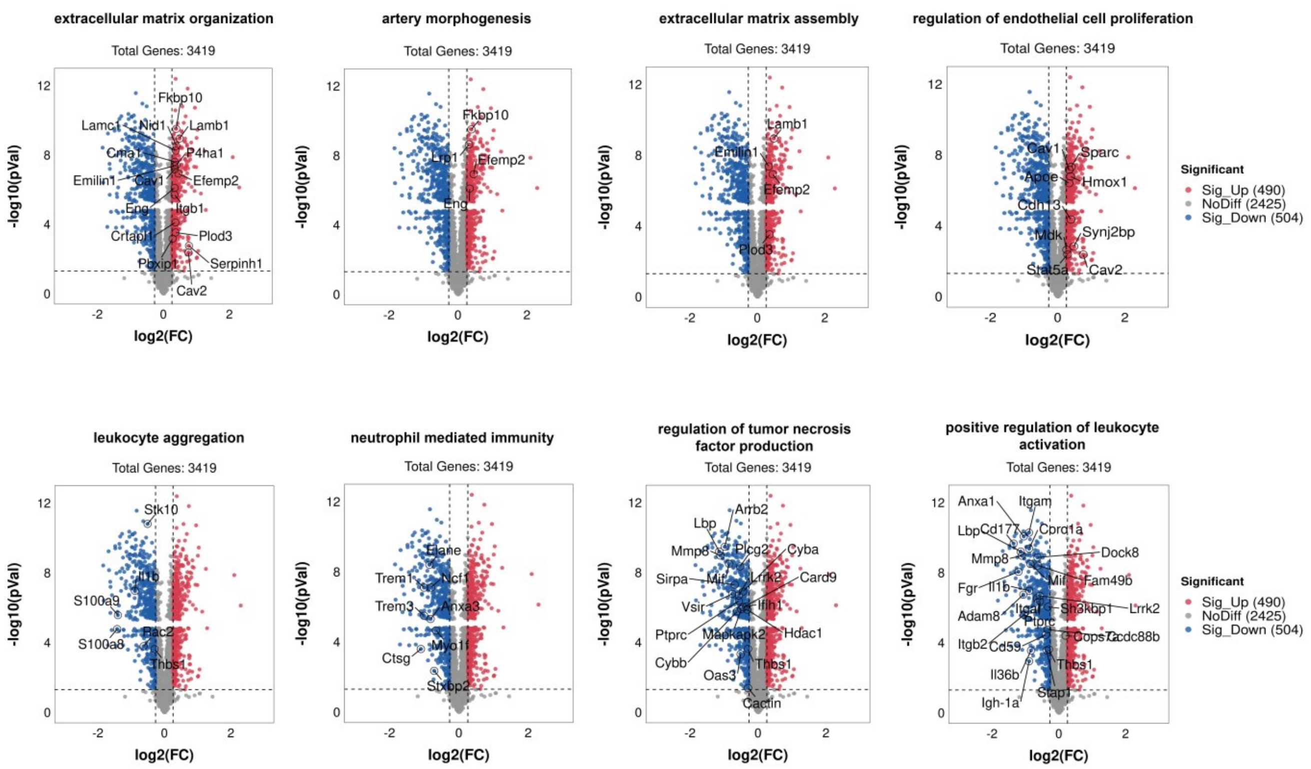


**Supplementary Figure 13.** The specific differentially expressed proteins related to wound healing in the biological process enrichment.

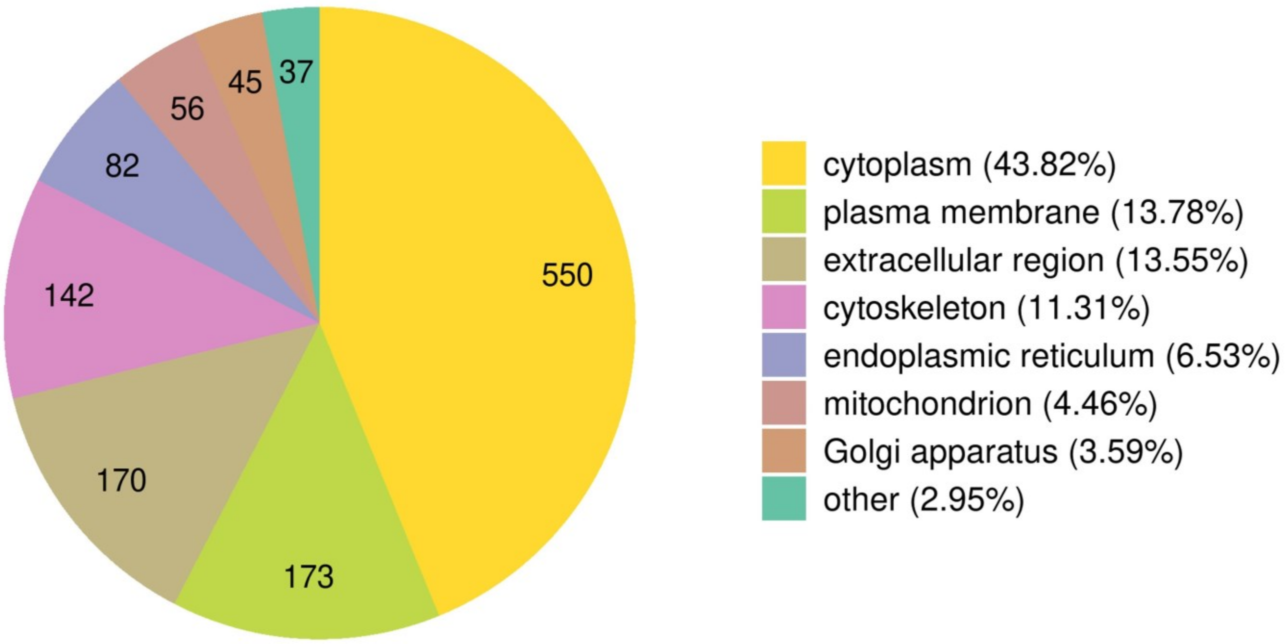


**Supplementary Figure 14.** The quantity and proportion of differentially expressed proteins in different organelles, in which the proteins in mitochondrion account for 4.46%.

**Supplementary Table 1.** Hydrogel samples with different formulas.

| Samples | PGA-SH | PGA-GMA | RGDC | LAP |
|---|---|---|---|---|
| Gel 1 | 4.5 w/v% | 4.5 w/v% | 0.1 w/v% | 0.1 w/v% |
| Gel 2 | 5.0 w/v% | 5.0 w/v% | 0.1 w/v% | 0.1 w/v% |
| Gel 3 | 5.5 w/v% | 5.5 w/v% | 0.1 w/v% | 0.1 w/v% |
| Gel 4 | 6.0 w/v% | 6.0 w/v% | 0.1 w/v% | 0.1 w/v% |

**Supplementary Table 2. Primer sequences*.**

| Gene | Forward | Reverse |
|---|---|---|
| *HIF-1a* | 5’-GGTGCTAACAGATGATGGTGAC-3’ | 5’-GGCTCATAACCCATCAACTCAG-3’ |
| *Tubulin* | 5’-TACCCTCGCATCCACTTCCCT-3’ | 5’-CGCTTGGTCTTGATGGTGGCA-3’ |

* Synthesized by General Bio Co., Ltd (Chuzhou, China).

## Supplementary References


1. Ghobril, C. & Grinstaff, M. W. The chemistry and engineering of polymeric hydrogel adhesives for wound closure: a tutorial. *Chem. Soc. Rev.* **44**, 1820-1835 (2015).
2. Yang, R. et al. Mechanoadaptive injectable hydrogel based on poly(gamma-glutamic acid) and hyaluronic acid regulates fibroblast migration for wound healing. *Carbohydr. Polym.* **273**, 118607 (2021).
3. Zhao, Y., Zhang, Y., Liu, A., Wei, Z. & Liu, S. Construction of Three-Dimensional Hemin-Functionalized Graphene Hydrogel with High Mechanical Stability and Adsorption Capacity for Enhancing Photodegradation of Methylene Blue. *Acs Appl. Mater. Interfaces*. **9**, 4006-4014 (2017).
4. Huang, J. et al. Programming electronic skin with diverse skin-like properties. *J. Mater. Chem. A*. **9**, 963-973 (2021).
