# Supplementary material for: A click chemistry-mediated all-peptide cell printing hydrogel platform for diabetic wound healing
Source: Nat Commun. 2023 Nov 29;14:7856. doi: 10.1038/s41467-023-43364-2 (PMC10687272; doi:10.1038/s41467-023-43364-2)
Supplement: Supplementary file 3 — Reporting Summary [file 41467_2023_43364_MOESM3_ESM.pdf]

nature portfolio

Corresponding author(s): Xiuwen Wu, Bo Chi, Jianan Ren

Last updated by author(s): Nov 6, 2023

Reporting Summary

Nature Portfolio wishes to improve the reproducibility of the work that we publish. This form provides structure for consistency and transparency in reporting. For further information on Nature Portfolio policies, see our Editorial Policies and the Editorial Policy Checklist.

Statistics

For all statistical analyses, confirm that the following items are present in the figure legend, table legend, main text, or Methods section.

n/a	Confirmed	
☐	☒	The exact sample size (n) for each experimental group/condition, given as a discrete number and unit of measurement
☐	☒	A statement on whether measurements were taken from distinct samples or whether the same sample was measured repeatedly
☐	☒	The statistical test(s) used AND whether they are one- or two-sided<br>*Only common tests should be described solely by name; describe more complex techniques in the Methods section.*
☒	☐	A description of all covariates tested
☐	☒	A description of any assumptions or corrections, such as tests of normality and adjustment for multiple comparisons
☐	☒	A full description of the statistical parameters including central tendency (e.g. means) or other basic estimates (e.g. regression coefficient) AND variation (e.g. standard deviation) or associated estimates of uncertainty (e.g. confidence intervals)
☐	☒	For null hypothesis testing, the test statistic (e.g. F, t, r) with confidence intervals, effect sizes, degrees of freedom and P value noted<br>*Give P values as exact values whenever suitable.*
☒	☐	For Bayesian analysis, information on the choice of priors and Markov chain Monte Carlo settings
☒	☐	For hierarchical and complex designs, identification of the appropriate level for tests and full reporting of outcomes
☒	☐	Estimates of effect sizes (e.g. Cohen's d, Pearson's r), indicating how they were calculated

Our web collection on statistics for biologists contains articles on many of the points above.

Software and code

Policy information about availability of computer code

Data collection	1. Material characterization: 1H NMR (Bruker 500, Germany), FTIR spectrometer (Nicolet-6700, Thermo Fisher, USA), SEM (LEO 1550, Germany), rheometer (DHR-2, TA-Instruments, USA), universal material testing machine (CMT2103, MTS, USA).<br>2. Cell imaging: white light microscope (CKX53, Olympus, Japan), fluorescence microscope (EVOS FLoid, Invitrogen, USA), confocal microscopy (LSM 980; ZEISS, Germany).<br>3. Flow cytometery: BD LSRFortessaTM X-20 flow cytometer.<br>4. Immune blot signaling detection: Tanon-4600 chemiluminescence imaging system (Tanon, Shanghai, China).<br>5. Mass spectrum: Orbitrap Exploris™ 480 mass spectrometer (Thermo Fisher Scientific, USA).<br>6. In vivo imaging: IVIS spectrum imaging system (XRMS Series III).<br>7. Vascularization imaging: LASCA using moorFLPI-2 (Moor Instruments, UK).<br>8. Tissue imaging: digital slide scanner (Pannoramic 250 FLASH, 3DHISTECH, Hungary).
Data analysis	1. GraphPad Prism 9 software was used to analyze statistical significance for all comparison studies using Student's t-test (unpaired and two-tailed), one-way ANOVA, or two-way ANOVA. Multiple comparisons were conducted.<br>2. ImageJ was used to analyse the signaling intensity.<br>3. Solidwork software was used to calculate the tangent angle of the adjacent planes in the side-view images of the printed cylinders.

For manuscripts utilizing custom algorithms or software that are central to the research but not yet described in published literature, software must be made available to editors and reviewers. We strongly encourage code deposition in a community repository (e.g. GitHub). See the Nature Portfolio guidelines for submitting code & software for further information.

Data

Policy information about availability of data

All manuscripts must include a data availability statement. This statement should provide the following information, where applicable:

- Accession codes, unique identifiers, or web links for publicly available datasets
- A description of any restrictions on data availability
- For clinical datasets or third party data, please ensure that the statement adheres to our policy

The proteomics data have been deposited at iProX (https://www.iprox.cn/page/home.html) with the dataset identifier PXD036045. Source data are provided with this paper. Data are available from the authors upon request.

Research involving human participants, their data, or biological material

Policy information about studies with human participants or human data. See also policy information about sex, gender (identity/presentation), and sexual orientation and race, ethnicity and racism.

Reporting on sex and gender	n/a
Reporting on race, ethnicity, or other socially relevant groupings	n/a
Population characteristics	n/a
Recruitment	n/a
Ethics oversight	n/a

Note that full information on the approval of the study protocol must also be provided in the manuscript.

Field-specific reporting

Please select the one below that is the best fit for your research. If you are not sure, read the appropriate sections before making your selection.

☒ Life sciences ☐ Behavioural & social sciences ☐ Ecological, evolutionary & environmental sciences

For a reference copy of the document with all sections, see nature.com/documents/nr-reporting-summary-flat.pdf

Life sciences study design

All studies must disclose on these points even when the disclosure is negative.

Sample size	Measurement of three samples in each group for hydrogel characterizations is a regular practice such as the examples reported in Nature Communications 12, 6070 (2021). Measurement of three samples in each group for cell experiments is also a routine practice such as the examples reported by Nature Biomedical Engineering 1, 983–992 (2017). More than 23 diabetic rats in each group with different hydrogel treatments exceeded the animal number reported in previous studies such as Science Advances 7, eabj0153 (2021) and Nature Communications 12, 3363 (2021).
Data exclusions	No data were excluded from the analyses.
Replication	All experiments were repeated and reliably reproduced. Number of rats used and independent experiments performed are indicated in the figure legends.
Randomization	Rats were randomly assigned to groups. Experimental units including mouse cages, culture wells were randomly organized in this study.
Blinding	All experiments and data collection was conducted as blinded including group allocation. All analyses were blinded to the experimental conditions.

Reporting for specific materials, systems and methods

We require information from authors about some types of materials, experimental systems and methods used in many studies. Here, indicate whether each material, system or method listed is relevant to your study. If you are not sure if a list item applies to your research, read the appropriate section before selecting a response.

Materials & experimental systems

n/a	Involved in the study	
☐	☒	Antibodies
☐	☒	Eukaryotic cell lines
☒	☐	Palaeontology and archaeology
☐	☒	Animals and other organisms
☒	☐	Clinical data
☒	☐	Dual use research of concern
☒	☐	Plants

Methods

n/a	Involved in the study	
☒	☐	ChIP-seq
☐	☒	Flow cytometry
☒	☐	MRI-based neuroimaging

Antibodies

Antibodies used

WB: Bax (1: 2000, ab32503, Abcam), Bcl2 (1: 2000, ab182858, Abcam), Cox iv (1: 2000, ab202554; Abcam), Cyt-c (1:5000, ab133504, Abcam), Cleaved casp-3 (1:500, ab32042, Abcam), Pro-casp-3 (1:1000, ab32150, Abcam), β-actin (1:1000, ab8226, Abcam), Goat anti-rabbit IgG (1:4000, ab6721, Abcam), Goat anti-mouse IgG (1:5000, ab205719, Abcam).

IF: VEGF antibody (1:50, ab155944, Abcam), anti-DNA/RNA damage antibody (1:200, ab183393, Abcam), CK10 (1:1000, GB111413, ServiceBio), CK14 (1:1000, GB11803, ServiceBio; 1:1000, MA5-11599, Invitrogen), CD31 (1:100, MA5-16951, Invitrogen), α-SMA (1:500, GB111364, ServiceBio), Ki67 (1:500, GB111141, ServiceBio), PGC-1α (1:100, ab106814, Abcam), Goat anti-mouse IgG Alexa Fluor® 647 (1:500, ab150115, Abcam), Goat anti-rabbit IgG Alexa Fluor® 488 (1:500, ab150077, Abcam), Goat anti-rabbit IgG Alexa Fluor® 647 (1:500, ab150083, Abcam), Donkey anti-goat IgG Cy3 (1:500, GB21404, ServiceBio).

Flow cytometery: CD31-FITC (1:100, ab33858, Abcam).

Validation

All the antibodies are commercially-available. The antibodies have been validated by the manufacturers, which can be found in the corresponding manufacturers' websites. No additional validation experiments were carried out.

Eukaryotic cell lines

Policy information about cell lines and Sex and Gender in Research

Cell line source(s)

HEK 293FT (SyngenTech, Beijing, China); HUVEC (P2-P4; Sciencell, USA).

Authentication

HEK 293FT cells were not authenticated. HUVECs were authenticated by IF staining of CD31.

Mycoplasma contamination

All cells were negative for mycoplasma.

Commonly misidentified lines (See ICLAC register)

None.

Animals and other research organisms

Policy information about studies involving animals; ARRIVE guidelines recommended for reporting animal research, and Sex and Gender in Research

Laboratory animals

For diabetic wounds without silicone ring restrictions: eighty-two male Sprague Dawley rats weighing almost 220 g at 7-8 weeks old were provided by Huachuang Sino Co., Ltd. The rats were housed in individual cages with free access to sterile water and chow, controlled temperature, and natural light–dark cycles. Three of them were randomly used as a non-diabetic control, and the other 79 rats were created with diabetic wounds followed by the independent treatment of the hydrogel (n=23), HUVECvector-laden hydrogel (n=28), or HUVECvegf165+-laden hydrogel (n=28).

For diabetic wounds with silicone ring restrictions: except wound restrictions by silicone ring, the methods of animal care, creating diabetic wounds, and interventions were the same for a total of 30 male Sprague Dawley rats at 7-8 weeks old with 10 rats in each group (n=10).

Wild animals

None.

Reporting on sex

Male.

Field-collected samples

None.

Ethics oversight

The animal protocol was approved by the Animal Investigation Ethics Committee of Jinling Hospital (2020JLHSKJDWLS-123).

Note that full information on the approval of the study protocol must also be provided in the manuscript.

Flow Cytometry

Plots

Confirm that:

☒ The axis labels state the marker and fluorochrome used (e.g. CD4-FITC).

☒ The axis scales are clearly visible. Include numbers along axes only for bottom left plot of group (a 'group' is an analysis of identical markers).

☒ All plots are contour plots with outliers or pseudocolor plots.

☒ A numerical value for number of cells or percentage (with statistics) is provided.

Methodology

Sample preparation	The regenerated granulation tissues of three rats in each group were harvested and stored in the sCelLiveTM Tissue Preservation Solution (Singleron, China) on ice. The specimens were washed with Hanks Balanced Salt Solution (HBSS) for three times, minced into small pieces, and then digested with 3 mL sCelLiVETM Tissue Dissociation Solution by Singleron PythoN™ Tissue Dissociation System at 37 °C for 15 min. The cell suspension was collected and filtered through a 40-micron sterile strainer. Afterwards, the GEXSCOPE® red blood cell lysis buffer (Singleron) was added, and the mixture [Cell: RCLB = 1:2 (volume ratio)] was incubated at room temperature for 5 min to remove red blood cells. The mixture was then centrifuged at 300 × g for 5 mins to remove supernatant and suspended softly with PBS. It was found by cell counting that around 600,000 cells were finally obtained for each group, in which 100,000 cells were isolated for cell staining with the vascular endothelial cell marker, CD31-FITC (ab33858; Abcam) and the mitochondrial damage marker, MitoSOX Red (HY-D1055; MCE) in turn according to the manufacturers' instruction.
Instrument	BD LSRFortessaTM X-20 flow cytometer.
Software	FlowJo 10.8.1.
Cell population abundance	Flow sorting of cells was not involved in this study.
Gating strategy	Different groups in which cells without dye staining, cells stained merely by CD31-FITC, and cells stained merely by MitoSOX Red were used in the gating strategy.

☒ Tick this box to confirm that a figure exemplifying the gating strategy is provided in the Supplementary Information.
